# Conflict between Noise and Plasticity in Yeast

**DOI:** 10.1371/journal.pgen.1001185

**Published:** 2010-11-04

**Authors:** Ben Lehner

**Affiliations:** Genetic Systems Laboratory, EMBL-CRG Systems Biology Unit and ICREA, CRG, UPF, Barcelona, Spain; University of Washington, United States of America

## Abstract

Gene expression responds to changes in conditions but also stochastically among individuals. In budding yeast, both expression responsiveness across conditions (“plasticity”) and cell-to-cell variation (“noise”) have been quantified for thousands of genes and found to correlate across genes. It has been argued therefore that noise and plasticity may be strongly coupled and mechanistically linked. This is consistent with some theoretical ideas, but a strong coupling between noise and plasticity also has the potential to introduce cost–benefit conflicts during evolution. For example, if high plasticity is beneficial (genes need to respond to the environment), but noise is detrimental (fluctuations are harmful), then strong coupling should be disfavored. Here, evidence is presented that cost–benefit conflicts do occur and that they constrain the evolution of gene expression and promoter usage. In contrast to recent assertions, coupling between noise and plasticity is not a general property, but one associated with particular mechanisms of transcription initiation. Further, promoter architectures associated with coupling are avoided when noise is most likely to be detrimental, and noise and plasticity are largely independent traits for core cellular components. In contrast, when genes are duplicated noise–plasticity coupling increases, consistent with reduced detrimental affects of expression variation. Noise–plasticity coupling is, therefore, an evolvable trait that may constrain the emergence of highly responsive gene expression and be selected against during evolution. Further, the global quantitative data in yeast suggest that one mechanism that relieves the constraints imposed by noise–plasticity coupling is gene duplication, providing an example of how duplication can facilitate escape from adaptive conflicts.

## Author Summary

Gene expression needs to respond to changes in conditions, but also varies stochastically among cells in a homogenous environment. It has been argued that these two levels of expression variation may be coupled, relating to the same underlying molecular mechanisms. However, such a strong coupling between expression “plasticity” and expression “noise” may introduce cost–benefit conflicts during evolution. For example, if plasticity is beneficial, but noise is detrimental, then coupling will be disfavored. In this work, evidence is presented that such cost–benefit conflicts do occur and that they constrain the evolution of gene expression in yeast. In contrast to recent conclusions, it is shown that noise–plasticity coupling is not a general result, but rather one associated with particular mechanisms of transcription initiation. Promoter architectures associated with coupling are avoided when noise is detrimental, and noise and plasticity are not coupled for core cellular components. Noise–plasticity coupling is therefore not a general property of gene expression, but an evolvable trait that may constrain the evolution of gene expression and be selected against during evolution. Further, gene duplication may facilitate escape from the adaptive conflict imposed by coupling.

## Introduction

For cellular adaptation gene expression must respond to changes in conditions. However, expression also varies stochastically among cells in a population. In the budding yeast *Saccharomyces cerevisiae* both variation across different conditions (‘expression plasticity’, [1]) and variation among individuals in a constant environment (‘expression noise’, [2]) have been quantified for thousands of genes. Comparisons across genes have shown that these two levels of expression correlate, suggesting that noise and plasticity may somehow be mechanistically coupled [3]–[7].

Correlations among different levels of expression variance are consistent with some theoretical proposals, which consider that stochastic, environmental, and mutational perturbations are likely to similarly affect biological systems [8]–[11]. The findings are also supported by mechanistic studies where mutations that increase noise have also increased plasticity [12], [13]. Further, several properties such as initiation from a TATA-box promoter [2], [4], [6] and high proximal promoter nucleosome occupancy [3], [14], [15] are enriched among genes with both high noise and high plasticity. It should be noted, however, that both properties are also associated with genes with a wide range of noise and plasticity levels [2]–[4], [6], [14], [15].

Theoretical work and intuition do, however, also suggest that noise and plasticity may not always by strongly coupled in this way. For example, by altering the size of transcriptional bursts, the interval time between bursts, or the number of decay steps in the degradation of a protein it should be possible to alter noise independently of plasticity [16]. Therefore it is important to ask whether coupling between expression noise and expression plasticity is, as reported [3], a general result. Or, rather, is coupling an evolvable trait that can vary among genes? Further, what is the mechanistic basis of coupling? Does coupling constrain expression evolution? How are such constraints relieved? And has coupling itself been subject to selection?

In some specific situations a strong coupling between noise and plasticity may be disfavored. For example, if high expression plasticity is beneficial, facilitating environmental adaptation, but high noise is detrimental, then strong coupling will cause a fitness cost–benefit conflict. This potential for an adaptive conflict predicts that for many genes strong coupling between noise and plasticity would reduce fitness. In short, when noise is detrimental, noise–plasticity coupling should be disfavored. It is not known if this is the case.

Here using global quantitative data from yeast it is shown that noise–plasticity coupling is not a general result, but rather a property of particular promoter architectures, and so is an evolvable trait. Promoter architectures that favor coupling are underrepresented among genes required for viability, and for these genes noise and plasticity are rather independent, consistent with selection against coupling. Following gene duplication, however, when the detrimental effects of expression variation in many cases will be reduced, the constraints on coupling and promoter architectures that favor coupling appear to be relaxed. Thus, noise–plasticity coupling is not a general trait, but one that likely is both constrained by selection and constrains the evolution of gene expression. Further, this constraint may be relieved following gene duplication, providing an example of escape from adaptive conflict [17], [18].

## Results

### Noise–plasticity coupling is related to promoter architecture

**Figure 1:**
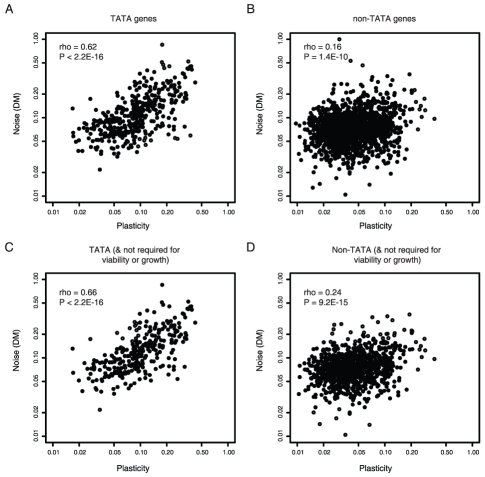
Noise–plasticity coupling relates to promoter architecture. Noise–plasticity coupling for genes initiating from TATA-box promoters (A) and non-TATA promoters (B). Scaled noise and plasticity levels are shown for all genes with available data in yeast. In (C,D) the same comparison is made, but excluding all essential genes, haploinsufficient genes and genes required for growth (C–D). Correlation coefficients and P-values are shown inset.

Considering all genes in yeast there is a reasonable correlation between levels of gene expression variation in a single condition (‘expression noise’ [2]) and the variability of expression across changing conditions (‘expression plasticity’ [1]) (Spearman's correlation coefficient, rho = 0.30, p<2.2E-16, n = 2049 [3]–[6]). However, considering different classes of genes shows that this is not a general result. In yeast only about 20% of genes are transcribed from promoters containing TATA box elements [19]. For these genes, noise and plasticity are strongly coupled (rho = 0.62, p<2.2E-16, n = 369, Figure 1A). In contrast, for non-TATA genes coupling is much weaker (rho = 0.16, p = 1.4E-10, n = 1680, Figure 1B). This indicates that the extent of coupling may relate to the mechanism of transcription initiation.

The stronger coupling of TATA genes is confirmed when controlling for possible confounding features such as the absolute level of plasticity (Table S1), the requirement of a gene for viability (Figure 1C and 1D), gene function (Table S3), expression level (Table S4), protein complex membership (Table S5), the number of upstream regulators (Table S6), the identity of upstream regulators (Table S7), histone exchange rates (Table S8), and nucleosome occupancy (Table 1). In summary, coupling between noise and plasticity in yeast appears related to the promoter architecture of a gene.

**Table 1:** Coupling between expression noise and expression plasticity for different gene classes in yeast.

Gene class	Spearman correlationcoefficient (rho)	P-value	Genes
Other genes (not e,h,s)	0.42	<2.2E-16	1281
Essential (e)	0.15	1.0E-03	492
Haploinsufficient (h)	−0.14	0.19	92
Slow growth (s)	0.05	0.43	262
TATA promoter	0.62	<2.2E-16	369
non-TATA promoter	0.16	1.4E-10	1680
TATA and not e,h,s	0.66	<2.2e-16	273
non-TATA and not e,h,s	0.24	9.2E-15	1009
Nucleosome occupied promoter	0.48	<2.2E-16	422
Nucleosome free promoter	0.13	2.4E-04	789
Nucleosome occupied and not e,h,s	0.59	<2.2E-16	308
Nucleosome free and not e,h,s	0.22	3.8E-06	442
Nucleosome occupied and not TATA	0.24	9.1E-05	268
Nucleosome free and not TATA	0.08	2.6E-02	709
Nucleosome occupied and TATA	0.57	<2.2E-16	154
Nucleosome free and TATA	0.46	1.7E-05	80

### Noise–plasticity coupling and chromatin dynamics

**Figure 2:**
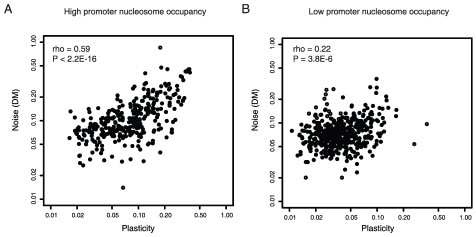
Noise–plasticity coupling is associated with promoter chromatin structure. Noise–plasticity coupling for genes with high proximal promoter nucleosome occupancy [15] (A) and low proximal promoter nucleosome occupancy (B). Here only genes not required for growth or viability are considered. The comparison for all genes and for genes transcribed from non-TATA promoters is shown in Figure S1. Spearman correlation coefficients and P-values are shown inset.

In eukaryotes DNA is packaged into chromatin, and this chromatin structure varies across the promoters of different genes [14], [15], [20]. Chromatin remodeling is thought to be a major source of transcriptional noise [13], [21], [22]. Many genes in yeast contain a DNA-encoded region of low nucleosome occupancy in their proximal promoters, a feature often associated with low expression noise [14], [15]. Considering the nucleosome occupancy of promoters shows that high noise–plasticity coupling is also associated with high upstream nucleosome occupancy (Figure S1A and S1B, rho = 0.13, p = 2.4E-4, n = 789 for genes with upstream nucleosome free regions and rho = 0.48, p<2.2E-16, n = 273 for genes with high upstream nucleosome occupancy). This result is confirmed when controlling for gene importance (Figure 2A and 2B), the absolute levels of plasticity (Table S1), and also when only considering non-TATA promoters, even though these promoters show lower overall levels of coupling (Table 1, rho = 0.08 for non-TATA genes with upstream nucleosome free regions and rho = 0.24 for non-TATA genes with high upstream nucleosome occupancy).

Promoters also differ in their nucleosome dynamics, and the exchange of core histones has been quantified across much of the genome [23]–[25]. High-noise plasticity coupling is also associated with high rates of histone exchange in promoter regions (Table S8). This is confirmed when controlling for TATA presence (Table S8), nucleosome occupancy (Table S9), or when only considering genes with low plasticity (Table S10). Thus, in addition to the link between noise–plasticity coupling and initiation from a TATA box, stronger coupling is also associated with higher and more dynamic promoter nucleosome occupancy. This strengthens the evidence that noise–plasticity coupling relates to the process of transcription initiation and indicates that coupling relates to chromatin remodeling. It also suggests that the extent of coupling is a trait that has the potential to change during evolution.

### Noise–plasticity coupling is disfavored for essential genes

High expression variation can be detrimental if it results in insufficient protein production, and there is good evidence that this is the case in yeast because genes required for viability have low noise [2], [5], [26], [27]. This predicts that noise–plasticity coupling should also be disfavored for these genes: although genes required for viability still need to respond to external conditions (for example coupling growth rates to changes in the environment), excessive variation in their production would be detrimental.

**Figure 3:**
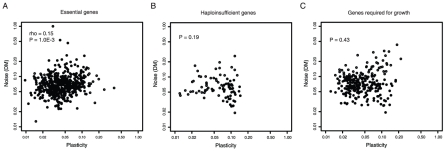
Low noise–plasticity coupling for core cellular components. The correlation between gene expression noise and gene expression plasticity is shown for essential genes (A), haploinsufficient genes (B), and genes required for growth (C). Noise and plasticity data are scaled between 0 and 1. Spearman rank correlation coefficients (rho) and P-values are shown for each gene class.

Consistent with this, essential genes, haploinsufficient genes (genes that reduce fitness when their copy number is reduced by half) and genes required for growth all show little or no significant coupling between noise and plasticity (Figure 3, rho = 0.15 p = 0.001 n = 492 for essential genes, p = 0.19 for haploinsufficient genes, and p = 0.43 for genes required for growth). Similar results are seen when only considering genes with low absolute levels of plasticity (Table S1). Consistent with predictions that noise will be detrimental for protein complex subunits [27], coupling is also lower for these genes (Table S2). Thus when high noise is likely to be detrimental, noise and plasticity are largely unrelated traits in yeast.

These findings are also consistent with differences in promoter architectures. Whereas 24% of non-essential genes use TATA promoters, only 3% of haploinsufficient genes, 9% of essential genes, and 11% of genes required for growth do (p<10E-14 in all cases, Fisher's exact test). Further, genes required for growth or viability usually have nucleosome free regions in their proximal promoters (74% compared to 57% for other genes, p<2.2E-16) and they have low levels of promoter histone exchange (mean 0.9 compared to 1.1 for other genes, p = 1.2E-11, Kolmogorov-Smirnov (KS) test). Thus, for genes encoding core cellular components, promoter architectures associated with high noise–plasticity coupling are avoided. This is consistent with a model in which selection disfavors coupling when it is detrimental.

### Increased noise–plasticity coupling following gene duplication

In many cases, therefore, the evolution of highly responsive gene expression from TATA promoters may be constrained by the detrimental effects of high noise. How can genes escape this adaptive conflict and evolve highly plastic TATA-initiating expression? One event that has been proposed as a general mechanism to facilitate escape from adaptive conflicts is gene duplication [17], [18]. Here it is argued that one conflict that can be resolved by duplication is the conflict between gene expression noise and plasticity.

Following the duplication of a gene, variation in expression can be less detrimental if there is functional compensation between duplicates and a component of the expression variation of the duplicates is independent [28]–[30]. Thus, if the evolution of highly responsive, but noisy, expression is constrained, then this constraint may be relieved by duplication: promoter architectures that favor plasticity (but that also couple this plasticity to noise) should be less detrimental. Three sets of observations from yeast are consistent with this proposal.

**Figure 4:**
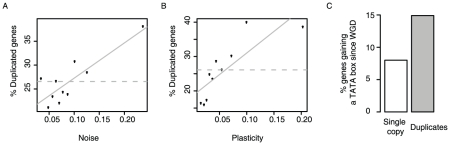
Gene duplicates are enriched amongst genes with the highest expression noise and plasticity, and they tend to gain TATA promoters. The proportion of duplicates is shown for genes with different expression noise (A) and plasticity (B). Proportions shown for equally populated bins of genes. (C) Genes retained as duplicates following the whole genome duplication in yeast are more likely to have gained a TATA box in their promoters since the duplication event than those reverting to a single copy (P = 1.24×10^−5^, Fischer's exact test). Here, only genes inferred to have ancestral TATA-less promoters are considered, N = 470 (retained as duplicates) and N = 1994 (not retained as duplicates).

First, duplicates in yeast have high levels of plasticity, and also high levels of noise (Figure 4A and 4B). Even duplicates known to redundantly perform a process required for viability or growth [31] have higher noise than single copy genes (p = 2.8E-7, KS test), showing that variation in their expression is not detrimental. Thus duplicates tolerate higher expression variation than other genes.

Second, TATA initiation, which facilitates plasticity but also couples noise and plasticity, is much more frequent among duplicates than among other genes: whereas 9% of genes required for viability or growth initiate from TATA promoters, this rises to 35% of gene duplicates redundantly required for growth or viability (p<2.2E-16, Fisher's exact test). TATA promoters are indeed strongly enriched among duplicates of all ages, and for duplicates arising from both small scale and whole genome duplication events (Table S11). Similar trends for the enrichment of TATA dependent transcription among duplicates are also seen in other species [32]. Thus, duplicates in general more frequently use promoters that couple noise to plasticity.

Third, evidence from the whole genome duplication (WGD) also supports this model. Following the WGD ∼100 million years ago, most genes reverted to a single copy but with a substantial number retained as duplicates [33]. This allows a direct comparison between genes retained as duplicates and those reverting to a single copy after a common ancestral event. Considering a set of genes inferred to have non-TATA promoters prior to the WGD (see Materials and Methods), those retained as duplicates are twice as likely to have gained a TATA promoter since the WGD than those reverting to a single copy (Figure 4C, P = 1.24×10^−5^, Fischer's exact test, N = 470 and N = 1994, respectively). This shows that not only are duplicates enriched for TATA promoters, but that they also tend to gain TATA promoters post-duplication. Thus, following duplication noise–plasticity coupling likely increased for many genes, which is consistent with this coupling being less detrimental.

In summary, the global data in yeast are consistent with duplication relieving a constraint on the evolution of highly plastic but variable gene expression. Thus one benefit of duplication may be that it allows escape from the adaptive conflict [17], [18] of noise–plasticity coupling.

## Discussion

### Noise–plasticity coupling is an evolvable trait

Previous studies have suggested that each promoter may have a “unique capacity to respond to external signals that can be environmental, genetic or even stochastic” [3]. Here it has been shown that this conclusion is not correct, but that the extent of noise–plasticity coupling relates to the mechanism of transcription initiation, and is confined to a subset of genes in yeast. This means that coupling can be an evolvable trait, as changes in promoter architecture associate with stronger or weaker coupling between expression plasticity and expression noise.

### Cost–benefit conflicts constrain the evolution of promoters and gene expression

This study initiated from the hypothesis that the reported strong coupling between noise and plasticity could be detrimental because of the potential for fitness cost–benefit conflicts. The quantitative data from yeast support this idea, showing that both noise–plasticity coupling and promoter architectures that favor coupling are avoided when coupling is likely to be detrimental. Although only correlative in nature, the data are consistent with noise–plasticity coupling being not just an evolvable trait, but also one that has likely been constrained by selection.

### High noise as a by-product of high plasticity for TATA genes

It has been shown here that TATA genes show a striking coupling between noise and plasticity. Thus, when high plasticity is adaptive, for TATA genes this will nearly always be accompanied by high noise. This means that although in some instances high noise may be beneficial [34]–[37], this should not be assumed as the case. Provided that it is not detrimental, high noise may be nothing more than a by-product of high plasticity.

### Gene duplication facilitates escape from the adaptive conflict of noise–plasticity coupling

For many genes, however, there is good evidence that high noise would be detrimental [2], [5],[26],[27] and for these genes TATA-dependent initiation is disfavored and strong noise–plasticity coupling is not observed. How can genes escape a potential adaptive conflict between the benefits of plasticity and the costs of noise? One likely mechanism is gene duplication. Whereas prior to duplication the detrimental consequences of noise may limit the evolution of highly responsive expression, following duplication variation may be better tolerated due to functional compensation [28]–[30]. Constraints on the evolution of highly responsive, but noisy expression should therefore be relieved following duplication. The quantitative data from yeast are consistent with this model, showing that noise, plasticity, and the use of TATA promoters all increase among duplicates. Thus one general benefit of duplication may be that it facilitates escape from the adaptive conflict [17], [18] imposed by coupling between expression noise and expression plasticity, permitting the evolution of responsive and variable expression.

## Materials and Methods

### Gene expression plasticity

Expression plasticity is defined as the total responsiveness of each gene's expression to environmental change in a large compendium of over 1500 *S. cerevisiae* expression profiling experiments [1], as reported in [6]. Values shown in this manuscript, as for noise, are scaled between 0 and 1.

### Gene expression noise

Expression noise is quantified from single cell-profiling measurements of fluorescently tagged proteins, using the ‘DM’ measure of Newman et al., which accounts for the influence of protein abundance on coefficient of variation measurements [2].

### Nucleosome occupancy and histone turnover

Promoters were classified as ‘nucleosome occupied’ or ‘nucleosome free’ using *in vivo* nucleosome occupancy data [38] in 100 base pairs upstream of each gene as previously described [15]. A total of 1082 ‘nucleosome occupied’ proximal promoters (clusters 7 and 8 from Tirosh et al.) and 1940 ‘nucleosome free’ proximal promoters (clusters 2, 3, and 4) are considered. Histone H3 exchange data is from [25]. The average exchange in each promoter is used, with a promoter defined as 500 base pairs upstream of a gene's start site.

### TATA box promoters

TATA containing promoters in *S. cerevisiae* were identified using the classification of Basehoar et al. [19]. Ancestral genes were considered as the set of genes with an ortholog present in each of the closely related pre-WGD species *Zygosaccharomyces rouxii, Kluyveromyces lactis, Ashbya gossypii, Saccharomyces kluyveri, Kluyveromyces thermotolerans, and Kluyveromyces waltii* [39]. TATA-boxes were identified in promoter regions of these species using the definition of Basehoar et al. by scanning the −70 to −310 region of each gene's promoter for the consensus site TATA(A/T)A(A/T)(A/G) [19]. In the analysis, ancestral non-TATA genes are those inferred from the absence of a consensus TATA-box in any pre-WGD species.

### Gene duplicates

Duplicates were identified using the SYNERGY algorithm [40], which uses gene trees based on sequence similarity and shared gene order across 17 fungal genomes to resolve orthology and paralogy relationships [41].

### Whole-genome duplicates

Whole-genome duplicates (WGD) and their orthologs in pre-WGD species were identified using the yeast genome order browser [42] version 3.0 [39]. Here conserved synteny and parsimony are used to identify ortholog groups.

### Genetic redundancy

Genetically redundant genes were compiled from systematic studies and the literature, as described [31]. Here only redundant genes where the single gene deletions do not result in slow growth are considered. Considering all redundant genes or redundant genes arising in the WGD gave very similar results.

All statistical tests were performed using R (www.r-project.org).

## Supporting Information

Figure S1 Noise-plasticity coupling is associated with high promoter nucleosome occupancy. The coupling between noise and plasticity for all genes with high (A) or low (B) proximal nucleosome occupancy. Correlation coefficients and P-values are shown inset.
(2.51 MB PDF)

Table S1 Correlation between expression noise and expression plasticity in yeast for different gene sets, only considering genes with normalized plasticity ≤ 0.1.
(0.03 MB DOC)

Table S2 Correlations between expression noise and expression plasticity in yeast for protein complex subunits.
(0.03 MB DOC)

Table S3 Plasticity-noise coupling for genes with different functions.
(0.11 MB DOC)

Table S4 Plasticity-noise coupling for genes with different expression levels.
(0.04 MB DOC)

Table S5 Plasticity-noise coupling for MIPs protein complex subunits and non-subunits, accounting for TATA status.
(0.03 MB DOC)

Table S6 Spearman correlation coefficients between noise (DM) and plasticity are shown for genes with different numbers of upstream regulators as determined by ChIP-chip (p<0.005, cons0 dataset).
(0.03 MB DOC)

Table S7 Plasticity-noise coupling for transcription factor targets.
(0.10 MB DOC)

Table S8 Plasticity-noise coupling for TATA genes with different promoter histone exchange rates.
(0.04 MB DOC)

Table S9 Plasticity-noise coupling for genes with different promoter histone exchange rates and nucleosome occupancies.
(0.03 MB DOC)

Table S10 Plasticity-noise coupling for genes with different promoter histone exchange rates and normalized plasticity ≤ 0.1.
(0.03 MB DOC)

Table S11 TATA genes are enriched among both ancestral and recent duplicates.
(0.03 MB DOC)
