# Supplementary figures and images for: Conflict between Noise and Plasticity in Yeast
Source: PLoS Genet. 2010 Nov 4;6(11):e1001185. doi: 10.1371/journal.pgen.1001185 (PMC2973811; doi:10.1371/journal.pgen.1001185)

Supplementary figure 1.

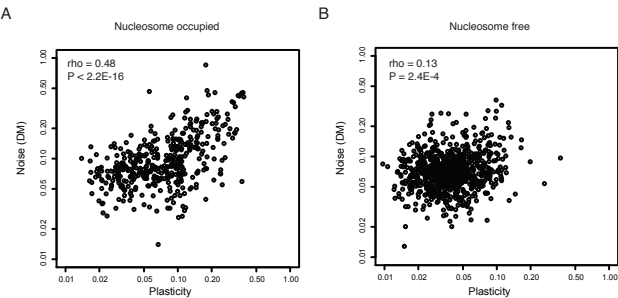

Supplement: Figure S1 — Noise-plasticity coupling is associated with high promoter nucleosome occupancy. The coupling between noise and plasticity for all genes with high (A) or low (B) proximal nucleosome occupancy. Correlation coefficients and P-values are shown inset. (2.51 MB PDF) [file pgen.1001185.s001.pdf]
