# Supplementary material for: Conflict between Noise and Plasticity in Yeast
Source: PLoS Genet. 2010 Nov 4;6(11):e1001185. doi: 10.1371/journal.pgen.1001185 (PMC2973811; doi:10.1371/journal.pgen.1001185)
Supplement: Table S1 — Correlation between expression noise and expression plasticity in yeast for different gene sets, only considering genes with normalized plasticity ≤ 0.1. (0.03 MB DOC) [file pgen.1001185.s002.doc]

**Table S1. Correlation between expression noise and expression plasticity in yeast for different gene sets, only considering genes with normalized plasticity ≤0.1.**

| **Gene class** | **Spearman correlation**  **coefficient (Rho)** | **P-value** | **Number of genes considered** |
| --- | --- | --- | --- |
|  |  |  |  |
| TATA promoter | 0.42 | 1.0E-10 | 225 |
| non-TATA promoter | 0.11 | 9.7E-6 | 1530 |
|  |  |  |  |
| High proximal promoter nucleosome occupancy | 0.24 | 5.8E-5 | 274 |
| Low proximal promoter nucleosome occupancy | 0.10 | 0.0057 | 760 |
|  |  |  |  |
| Essential (e) | 0.11 | 0.019 | 455 |
| Haploinsufficient (h) | -0.057 | 0.64 | 68 |
| Slow growth (s) | 0.0075 | 0.91 | 233 |
| Other (not e,h,s) | 0.25 | <2.2E-16 | 1059 |
