# Supplementary material for: Conflict between Noise and Plasticity in Yeast
Source: PLoS Genet. 2010 Nov 4;6(11):e1001185. doi: 10.1371/journal.pgen.1001185 (PMC2973811; doi:10.1371/journal.pgen.1001185)
Supplement: Table S2 — Correlations between expression noise and expression plasticity in yeast for protein complex subunits. (0.03 MB DOC) [file pgen.1001185.s003.doc]

**Table S2. Correlations between expression noise and expression plasticity in yeast for protein complex subunits.**

Spearman correlation coefficients between noise (DM) and plasticity are shown for genes that are subunits of MIPS literature-curated protein complexes.

|  | **MIPS complex subunits** | | | **Other** | | |
| --- | --- | --- | --- | --- | --- | --- |
| **Gene set** | **Rho** | **P-value** | **Genes** | **Rho** | **P-value** | **Genes** |
| All genes | 0.12 | 0.0039 | 560 | 0.37 | < 2.2e-16 | 1489 |
| Genes not required for viability or growth | 0.23 | 4.1E-05 | 322 | 0.43 | < 2.2e-16 | 1196 |
| Genes required for viability or growth | -0.05 | 0.45 | 238 | 0.13 | 0.025 | 293 |
