# Supplementary material for: Conflict between Noise and Plasticity in Yeast
Source: PLoS Genet. 2010 Nov 4;6(11):e1001185. doi: 10.1371/journal.pgen.1001185 (PMC2973811; doi:10.1371/journal.pgen.1001185)
Supplement: Table S3 — Plasticity-noise coupling for genes with different functions. (0.11 MB DOC) [file pgen.1001185.s004.doc]

**Table S3. Plasticity-noise coupling for genes with different functions.**

Spearman correlation coefficients between noise (DM) and plasticity are shown for genes annotated with all GOslim terms with 10 or more TATA and non-TATA genes.

|  |  | **non-TATA promtoers** | | | **TATA promoters** | | |
| --- | --- | --- | --- | --- | --- | --- | --- |
| **GOslim** | **Description** | **Rho** | **P-value** | **Genes** | **Rho** | **P-value** | **Genes** |
| GO:0003674 | molecular_function | 0.18 | 1.5E-04 | 445 | 0.65 | 0.0000 | 80 |
| GO:0005198 | structural molecule activity | -0.37 | 0.0000 | 147 | 0.57 | 0.0280 | 15 |
| GO:0005215 | transporter activity | 0.37 | 0.0001 | 107 | 0.46 | 0.0016 | 46 |
| GO:0005515 | protein binding | 0.10 | 0.1859 | 173 | 0.54 | 0.0018 | 31 |
| GO:0005618 | cell wall | 0.14 | 0.6131 | 16 | 0.20 | 0.4778 | 15 |
| GO:0005624 | membrane fraction | 0.48 | 0.0030 | 37 | 0.20 | 0.5717 | 10 |
| GO:0005634 | nucleus | 0.14 | 0.0008 | 594 | 0.57 | 0.0000 | 85 |
| GO:0005737 | cytoplasm | 0.15 | 0.0000 | 1104 | 0.57 | 0.0000 | 248 |
| GO:0005739 | mitochondrion | 0.32 | 0.0000 | 283 | 0.48 | 0.0000 | 113 |
| GO:0005740 | mitochondrial envelope | 0.37 | 0.0016 | 71 | 0.58 | 0.0003 | 35 |
| GO:0005773 | vacuole | 0.34 | 0.0043 | 71 | 0.76 | 0.0013 | 15 |
| GO:0005783 | endoplasmic reticulum | 0.12 | 0.1419 | 147 | 0.39 | 0.0183 | 37 |
| GO:0005840 | ribosome | -0.29 | 0.0003 | 160 | 0.30 | 0.2510 | 16 |
| GO:0005886 | plasma membrane | 0.50 | 0.0000 | 79 | 0.51 | 0.0018 | 35 |
| GO:0005933 | cellular bud | 0.14 | 0.2394 | 68 | 0.30 | 0.2737 | 15 |
| GO:0005975 | carbohydrate metabolic process | 0.16 | 0.1962 | 71 | 0.66 | 0.0000 | 34 |
| GO:0006091 | generation of precursor metabolites and energy | 0.50 | 0.0004 | 46 | 0.24 | 0.1330 | 40 |
| GO:0006350 | transcription | -0.07 | 0.3190 | 186 | 0.76 | 0.0006 | 17 |
| GO:0006412 | translation | -0.32 | 0.0000 | 177 | 0.19 | 0.4638 | 17 |
| GO:0006457 | protein folding | -0.04 | 0.8443 | 22 | 0.68 | 0.0067 | 15 |
| GO:0006464 | protein modification process | -0.01 | 0.8484 | 186 | 0.38 | 0.1670 | 15 |
| GO:0006519 | cellular amino acid and derivative metabolic process | 0.21 | 0.0720 | 74 | 0.69 | 0.0000 | 36 |
| GO:0006629 | lipid metabolic process | 0.07 | 0.5322 | 76 | 0.26 | 0.1511 | 31 |
| GO:0006766 | vitamin metabolic process | 0.64 | 0.0054 | 18 | 0.82 | 0.0019 | 12 |
| GO:0006810 | transport | 0.25 | 0.0000 | 366 | 0.59 | 0.0000 | 76 |
| GO:0006950 | response to stress | 0.16 | 0.0463 | 147 | 0.68 | 0.0000 | 44 |
| GO:0006996 | organelle organization | 0.03 | 0.5331 | 481 | 0.46 | 0.0007 | 52 |
| GO:0007047 | cell wall organization | 0.09 | 0.5457 | 47 | 0.79 | 0.0001 | 19 |
| GO:0007049 | cell cycle | 0.06 | 0.5172 | 116 | 0.31 | 0.3251 | 12 |
| GO:0007165 | signal transduction | 0.31 | 0.0056 | 79 | 0.62 | 0.0361 | 12 |
| GO:0008150 | biological_process | 0.28 | 0.0001 | 194 | 0.67 | 0.0000 | 55 |
| GO:0012505 | endomembrane system | 0.16 | 0.0604 | 139 | 0.06 | 0.8265 | 16 |
| GO:0016020 | membrane | 0.29 | 0.0000 | 314 | 0.57 | 0.0000 | 73 |
| GO:0016044 | membrane organization | 0.30 | 0.0039 | 92 | 0.63 | 0.0312 | 12 |
| GO:0016070 | RNA metabolic process | 0.03 | 0.5619 | 387 | 0.59 | 0.0012 | 28 |
| GO:0016192 | vesicle-mediated transport | 0.12 | 0.1046 | 183 | 0.48 | 0.0442 | 18 |
| GO:0016491 | oxidoreductase activity | 0.43 | 0.0004 | 66 | 0.39 | 0.0010 | 69 |
| GO:0016740 | transferase activity | 0.12 | 0.0716 | 211 | 0.56 | 0.0000 | 51 |
| GO:0016787 | hydrolase activity | 0.15 | 0.0164 | 257 | 0.66 | 0.0000 | 46 |
| GO:0016829 | lyase activity | 0.50 | 0.0210 | 21 | 0.32 | 0.2452 | 15 |
| GO:0016874 | ligase activity | 0.14 | 0.3321 | 50 | 0.73 | 0.0061 | 13 |
| GO:0019725 | cellular homeostasis | 0.04 | 0.7863 | 47 | 0.80 | 0.0000 | 19 |
| GO:0030427 | site of polarized growth | 0.21 | 0.0328 | 101 | 0.10 | 0.7049 | 17 |
| GO:0042221 | response to chemical stimulus | 0.22 | 0.0177 | 118 | 0.55 | 0.0001 | 46 |
| GO:0042254 | ribosome biogenesis | 0.13 | 0.0605 | 220 | 0.13 | 0.6626 | 13 |
| GO:0045333 | cellular respiration | 0.08 | 0.7375 | 22 | 0.15 | 0.5748 | 16 |
| GO:0051186 | cofactor metabolic process | 0.40 | 0.0047 | 49 | 0.56 | 0.0038 | 25 |
