# Supplementary material for: Conflict between Noise and Plasticity in Yeast
Source: PLoS Genet. 2010 Nov 4;6(11):e1001185. doi: 10.1371/journal.pgen.1001185 (PMC2973811; doi:10.1371/journal.pgen.1001185)
Supplement: Table S4 — Plasticity-noise coupling for genes with different expression levels. (0.04 MB DOC) [file pgen.1001185.s005.doc]

**Table S4. Plasticity-noise coupling for genes with different expression levels.**

Spearman correlation coefficients between noise (DM) and plasticity are shown for genes divided into 5 approximately equally populated bins of genes according to their expression levels determined in Newman et al (2006).

|  | **non-TATA promoters** | | | **TATA promoters** | | |
| --- | --- | --- | --- | --- | --- | --- |
| **Expression level bin** | **Rho** | **P-value** | **Genes** | **Rho** | **P-value** | **Genes** |
| bin 1 (lowest expression) | 0.19 | 0.00019 | 368 | 0.58 | 1.0E-05 | 50 |
| bin 2 | 0.24 | 6.4E-06 | 345 | 0.70 | 9.0E-10 | 58 |
| bin 3 | 0.20 | 0.00019 | 339 | 0.68 | < 2.2e-16 | 72 |
| bin 4 | 0.28 | 4.0E-06 | 361 | 0.70 | < 2.2e-16 | 68 |
| bin 5 (highest expression) | 0.25 | 8.7E-07 | 368 | 0.61 | < 2.2e-16 | 121 |
