# Supplementary material for: Conflict between Noise and Plasticity in Yeast
Source: PLoS Genet. 2010 Nov 4;6(11):e1001185. doi: 10.1371/journal.pgen.1001185 (PMC2973811; doi:10.1371/journal.pgen.1001185)
Supplement: Table S5 — Plasticity-noise coupling for MIPs protein complex subunits and non-subunits, accounting for TATA status. (0.03 MB DOC) [file pgen.1001185.s006.doc]

**Table S5. Plasticity-noise coupling for MIPs protein complex subunits and non-subunits, accounting for TATA status.**

Spearman correlation coefficients between noise (DM) and plasticity are shown for genes that are subunits of MIPs literature-curated protein complexes or MIPs complexes including complexes defined from systematic studies.

|  | **non-TATA promoters** | | | **TATA promoters** | | |
| --- | --- | --- | --- | --- | --- | --- |
| **Gene set** | **Rho** | **P-value** | **Genes** | **Rho** | **P-value** | **Genes** |
| MIPS complex subunits | -0.02 | 0.676 | 489 | 0.63 | 3.14E-09 | 71 |
| not MIPS complex subunits | 0.23 | 3.61E-16 | 1191 | 0.64 | < 2.2e-16 | 297 |
| MIPS complex subunits including systematic complexes | 0.14 | 3.51E-06 | 1056 | 0.65 | < 2.2e-16 | 200 |
| not MIPS complex subunits or systematic complex subunits | 0.19 | 2.11E-06 | 624 | 0.59 | < 2.2e-16 | 168 |
