# Supplementary material for: Conflict between Noise and Plasticity in Yeast
Source: PLoS Genet. 2010 Nov 4;6(11):e1001185. doi: 10.1371/journal.pgen.1001185 (PMC2973811; doi:10.1371/journal.pgen.1001185)
Supplement: Table S6 — Spearman correlation coefficients between noise (DM) and plasticity are shown for genes with different numbers of upstream regulators as determined by ChIP-chip (p<0.005, cons0 dataset). (0.03 MB DOC) [file pgen.1001185.s007.doc]

**Table S6. Plasticity-noise coupling for genes with different numbers of upstream regulators.**

Spearman correlation coefficients between noise (DM) and plasticity are shown for genes with different numbers of upstream regulators as determined by ChIP-chip (p<0.005, cons0 dataset from MacIsaac et al. (2006)).

|  | **non-TATA promoters** | | | **TATA promoters** | | |
| --- | --- | --- | --- | --- | --- | --- |
| **Regulators** | **Rho** | **P-value** | **Genes** | **Rho** | **P-value** | **Genes** |
| 0 | 0.10 | 0.0182 | 572 | 0.63 | 9.47E-11 | 86 |
| 1 | 0.10 | 0.0430 | 399 | 0.57 | 1.14E-06 | 64 |
| 2 | 0.29 | 0.0000 | 254 | 0.65 | 2.22E-06 | 46 |
| 3-4 | 0.13 | 0.0374 | 243 | 0.59 | 4.03E-08 | 76 |
| 5+ | 0.28 | 0.0002 | 182 | 0.45 | 6.02E-06 | 95 |
