# Supplementary material for: Conflict between Noise and Plasticity in Yeast
Source: PLoS Genet. 2010 Nov 4;6(11):e1001185. doi: 10.1371/journal.pgen.1001185 (PMC2973811; doi:10.1371/journal.pgen.1001185)
Supplement: Table S7 — Plasticity-noise coupling for transcription factor targets. (0.10 MB DOC) [file pgen.1001185.s008.doc]

**Table S7. Plasticity-noise coupling for transcription factor targets.**

Spearman correlation coefficients between noise (DM) and plasticity are shown for genes that are targets of different transcription factors as determined by ChIP-chip (p<0.005, cons0 dataset from MacIsaac et al. (2006)).

|  | **non-TATA promoters** | | | **TATA promoters** | | |
| --- | --- | --- | --- | --- | --- | --- |
| **Transcription factor targets** | **Rho** | **P-value** | **Genes** | **Rho** | **P-value** | **Genes** |
| ABF1 | 0.17 | 0.026 | 166 | 0.35 | 0.048 | 32 |
| AFT2 | 0.31 | 0.037 | 47 | 0.55 | 0.005 | 25 |
| ASH1 | 0.54 | 0.091 | 11 | 0.18 | 0.547 | 13 |
| BAS1 | -0.03 | 0.923 | 15 | 0.52 | 0.072 | 13 |
| CBF1 | 0.48 | 0.000 | 95 | 0.45 | 0.012 | 30 |
| CIN5 | 0.38 | 0.004 | 55 | 0.59 | 0.001 | 28 |
| DAL82 | 0.21 | 0.148 | 48 | 0.79 | 0.009 | 10 |
| DIG1 | 0.38 | 0.001 | 72 | 0.39 | 0.163 | 14 |
| FHL1 | -0.16 | 0.163 | 75 | 0.50 | 0.083 | 13 |
| FKH1 | 0.30 | 0.019 | 60 | 0.73 | 0.007 | 12 |
| FKH2 | 0.50 | 0.000 | 57 | 0.81 | 0.000 | 16 |
| GCN4 | 0.48 | 0.000 | 71 | 0.46 | 0.003 | 40 |
| GCR2 | 0.24 | 0.175 | 33 | 0.70 | 0.000 | 24 |
| GLN3 | 0.62 | 0.000 | 39 | 0.62 | 0.001 | 26 |
| HAP1 | 0.29 | 0.072 | 40 | 0.25 | 0.201 | 27 |
| HAP2 | 0.60 | 0.000 | 35 | 0.45 | 0.168 | 11 |
| HAP4 | 0.07 | 0.712 | 27 | 0.18 | 0.586 | 11 |
| HAP5 | 0.53 | 0.004 | 29 | 0.41 | 0.148 | 14 |
| HSF1 | 0.21 | 0.420 | 16 | 0.45 | 0.168 | 11 |
| INO4 | 0.17 | 0.400 | 27 | 0.79 | 0.000 | 16 |
| MAC1 | 0.24 | 0.205 | 30 | 0.59 | 0.077 | 10 |
| MBP1 | 0.36 | 0.002 | 75 | 0.54 | 0.016 | 20 |
| MET31 | 0.04 | 0.848 | 21 | 0.66 | 0.007 | 15 |
| MOT3 | 0.16 | 0.281 | 49 | -0.02 | 0.952 | 16 |
| MSN2 | 0.31 | 0.011 | 68 | 0.50 | 0.000 | 54 |
| MSN4 | 0.16 | 0.223 | 59 | 0.76 | 0.000 | 39 |
| NDD1 | 0.57 | 0.000 | 39 | 0.73 | 0.001 | 17 |
| NRG1 | 0.44 | 0.008 | 36 | 0.55 | 0.003 | 27 |
| PHD1 | 0.47 | 0.001 | 51 | 0.57 | 0.010 | 20 |
| PHO2 | 0.10 | 0.332 | 91 | 0.37 | 0.032 | 34 |
| RAP1 | -0.20 | 0.045 | 99 | 0.23 | 0.442 | 13 |
| RCS1 | 0.35 | 0.019 | 44 | 0.27 | 0.324 | 15 |
| REB1 | 0.16 | 0.059 | 135 | 0.51 | 0.018 | 22 |
| ROX1 | 0.41 | 0.005 | 46 | 0.38 | 0.048 | 28 |
| RTG3 | 0.21 | 0.245 | 33 | 0.56 | 0.008 | 22 |
| SKN7 | 0.29 | 0.019 | 65 | 0.60 | 0.000 | 43 |
| SOK2 | 0.56 | 0.001 | 33 | 0.39 | 0.085 | 21 |
| STE12 | 0.32 | 0.001 | 96 | 0.46 | 0.033 | 22 |
| STP1 | 0.13 | 0.629 | 16 | 0.82 | 0.000 | 15 |
| SUT1 | 0.35 | 0.076 | 26 | 0.38 | 0.119 | 18 |
| SWI4 | 0.24 | 0.105 | 48 | 0.46 | 0.020 | 26 |
| SWI5 | 0.33 | 0.068 | 32 | 0.63 | 0.009 | 16 |
| SWI6 | 0.28 | 0.061 | 47 | 0.64 | 0.001 | 24 |
| TYE7 | 0.44 | 0.018 | 29 | 0.16 | 0.624 | 11 |
| UME6 | 0.14 | 0.300 | 56 | 0.68 | 0.000 | 26 |
| XBP1 | 0.78 | 0.004 | 12 | 0.35 | 0.218 | 14 |
| YAP1 | 0.42 | 0.067 | 20 | 0.59 | 0.030 | 14 |
| YAP6 | 0.37 | 0.015 | 42 | 0.48 | 0.015 | 25 |
| YAP7 | 0.32 | 0.017 | 56 | 0.46 | 0.025 | 24 |
