# Supplementary material for: Conflict between Noise and Plasticity in Yeast
Source: PLoS Genet. 2010 Nov 4;6(11):e1001185. doi: 10.1371/journal.pgen.1001185 (PMC2973811; doi:10.1371/journal.pgen.1001185)
Supplement: Table S8 — Plasticity-noise coupling for TATA genes with different promoter histone exchange rates. (0.04 MB DOC) [file pgen.1001185.s009.doc]

**Table S8. Plasticity-noise coupling for TATA genes with different promoter histone exchange rates.**

Spearman correlation coefficients between noise (DM) and plasticity for genes with different promoter histone H3 exchange rates. Genes are grouped into 5 approximately equally sized bins according to the mean exchange rates in 500 bp upstream of each gene’s start codon.

|  | **non-TATA promoters** | | | **TATA promoters** | | |
| --- | --- | --- | --- | --- | --- | --- |
| **Promoter histone exchange** | **Rho** | **P-value** | **Genes** | **Rho** | **P-value** | **Genes** |
| bin 1 – lowest exchange | 0.06 | 0.37 | 246 | 0.41 | 0.090 | 18 |
| bin 2 | 0.04 | 0.49 | 368 | 0.57 | 0.0005 | 34 |
| bin 3 | 0.16 | 0.002 | 400 | 0.52 | 1.41E-05 | 64 |
| bin 4 | 0.13 | 0.017 | 350 | 0.69 | < 2.2e-16 | 81 |
| bin 5 – highest exchange | 0.29 | 1.49E-07 | 316 | 0.60 | < 2.2e-16 | 172 |
