# Supplementary material for: Conflict between Noise and Plasticity in Yeast
Source: PLoS Genet. 2010 Nov 4;6(11):e1001185. doi: 10.1371/journal.pgen.1001185 (PMC2973811; doi:10.1371/journal.pgen.1001185)
Supplement: Table S9 — Plasticity-noise coupling for genes with different promoter histone exchange rates and nucleosome occupancies. (0.03 MB DOC) [file pgen.1001185.s010.doc]

**Table S9. Plasticity-noise coupling for genes with different promoter histone exchange rates and nucleosome occupancies.**

Spearman correlation coefficients between noise (DM) and plasticity for genes with different promoter histone H3 exchange rates. Genes are grouped into 5 approximately equally sized bins according to the mean exchange rates in 500 bp upstream of each gene’s start codon.

|  | **Low nucleosome occupancy** | | | **High nucleosome occupancy** | | |
| --- | --- | --- | --- | --- | --- | --- |
| **Promoter histone exchange** | **Rho** | **P-value** | **Genes** | **Rho** | **P-value** | **Genes** |
| bin 1 – lowest exchange | 0.06 | 0.46 | 144 | 0.39 | 0.12 | 17 |
| bin 2 | 0.03 | 0.70 | 214 | 0.53 | 1.4E-03 | 35 |
| bin 3 | 0.21 | 0.001 | 222 | 0.33 | 1.3E-02 | 56 |
| bin 4 | 0.15 | 0.083 | 132 | 0.29 | 0.01 | 89 |
| bin 5 – highest exchange | 0.24 | 3.3E-02 | 77 | 0.55 | < 2.2e-16 | 225 |
