# Supplementary material for: Conflict between Noise and Plasticity in Yeast
Source: PLoS Genet. 2010 Nov 4;6(11):e1001185. doi: 10.1371/journal.pgen.1001185 (PMC2973811; doi:10.1371/journal.pgen.1001185)
Supplement: Table S10 — Plasticity-noise coupling for genes with different promoter histone exchange rates and normalized plasticity ≤ 0.1. (0.03 MB DOC) [file pgen.1001185.s011.doc]

**Table S10. Plasticity-noise coupling for genes with different promoter histone exchange rates and normalized plasticity ≤0.1.**

Spearman correlation coefficients between noise (DM) and plasticity for genes with different promoter histone H3 exchange rates and normalized plasticity ≤0.1. Genes are grouped into 5 approximately equally sized bins according to the mean exchange rates in 500 bp upstream of each gene’s start codon.

|  | **Spearman correlation coefficient** | | |
| --- | --- | --- | --- |
| **Promoter histone exchange** | **Rho** | **P-value** | **Genes** |
| bin 1 – lowest exchange | 0.04 | 0.51 | 250 |
| bin 2 | 0.03 | 0.51 | 375 |
| bin 3 | 0.17 | 3.7E-04 | 419 |
| bin 4 | 0.21 | 6.1E-05 | 358 |
| bin 5 – highest exchange | 0.24 | 6.4E-06 | 353 |
