# Supplementary material for: Conflict between Noise and Plasticity in Yeast
Source: PLoS Genet. 2010 Nov 4;6(11):e1001185. doi: 10.1371/journal.pgen.1001185 (PMC2973811; doi:10.1371/journal.pgen.1001185)
Supplement: Table S11 — TATA genes are enriched among both ancestral and recent duplicates. (0.03 MB DOC) [file pgen.1001185.s012.doc]

**Table S11. TATA genes are enriched among both ancestral and recent duplicates.**

| **Gene set** | **% TATA-regulated genes** | **Genes** | **P-value^1^** |
| --- | --- | --- | --- |
| Single copy | 15% | 4253 |  |
|  |  |  |  |
| All duplicates | 28% | 1297 | < 2.2×10^-16^ |
|  |  |  |  |
| pre-WGD^2^ duplicates | 32% | 508 | < 2.2×10^-16^ |
| WGD duplicates | 26% | 876 | 3.86×10^-13^ |
| post-WGD duplicates | 38% | 218 | 5.04×10^-15^ |

^1^ P-value for difference from single copy genes (Fisher’s exact test).

^2^ WGD – whole genome duplication.
